# Self management for people with psychotic vulnerability: a feasibility study

**Published:** 2012-06-15

**Authors:** Rob de Leeuw, Ina Boerema

**Affiliations:** University Medical Center Utrecht, The Netherlands; Trimbos – Netherlands Institute of Mental Health and Addiction, The Netherlands

**Keywords:** self-management, schizophrenia, disease management, internet platform

## Abstract

**Introduction:**

Presentation of Project “Personal Control in Rehabilitation” (PCR: Self management for people with psychotic vulnerability): internet application with one public portal and three secured portals for patients, relatives and caregivers. Joint project of two Mental Health Institutes, Julius Center Utrecht University, Trimbos Institute and Vital Health Software. It is the first ICT-project specifically for this target group in the Netherlands, started October 2008. Pilot phase with 60 patients, their relatives and caregivers is ended in Spring 2011. Then more patients will participate, other Mental Health Institutes will join and new research is planned.

**Aims and objectives:**

Facilitating communication between people with psychotic disorders, their relatives/carers and professional caregivers. Giving access to information about their treatment, rehab and crisis plan, and to general information on possibilities for treatment and recovery. Stimulating shared decision-making and self-management. Better and quicker insight in the patient’s development. Less clinical and therefore cheaper care. By means of a secured internet-site with several communication and self management tools, information about individual care plans, experiences of peers, care possibilities, recovery strategies etc.

**Results:**

The pilot-phase resulted in a well-developed internet site; this will be shown during the presentation. The expectations based on a business case study will be shown together with the methods and the main results of the study.

**Conclusions:**

Positive conclusions will be drawn about the feasibility of the project, its suitability for people with psychotic vulnerability, their formal and informal caregivers, and about the economic effects. The preconditions for the efficacy of this internet-program will be presented. Further steps for use of PCR on a larger scale and for extensions of the program will be discussed.

